# Case Report: Marked Survival Advantage of Two Colorectal Cancer Patients with Liver Metastases Treated with Vigil and FOLFOX-6

**DOI:** 10.3390/vaccines9101201

**Published:** 2021-10-18

**Authors:** Vedin Barve, Ned Adams, Laura Stanbery, Luisa Manning, Staci Horvath, Gladice Wallraven, Ernest Bognar, Minal Barve, John Nemunaitis

**Affiliations:** 1 Texas Oncology Physician Association, Dallas, TX 75251, USA; vedin.barve@utexas.edu (V.B.); mbarve@marycrowley.org (M.B.); 2 Mary Crowley Cancer Research, Dallas, TX 75230, USA; NAdams@marycrowley.org; 3 Gradalis, Inc., Carrollton, TX 75006, USA; lnejedlik@gradalisinc.com (L.S.); lmanning@gradalisinc.com (L.M.); shorvath@gradalisinc.com (S.H.); gwallraven@gradalisinc.com (G.W.); ebognar@gradalisinc.com (E.B.)

**Keywords:** colorectal cancer, immunotherapy, liver metastases

## Abstract

Colorectal cancer is the third most diagnosed cancer in the United States. Five-year survival rates remain low and many patients will develop liver metastasis. Vigil is an immunotherapy manufactured from autologous tumor cells and transfected ex vivo with a plasmid that encodes the GM-CSF gene and bifunctional shRNA construct to knockdown furin. Here, we report two patients with colorectal cancer and resectable liver metastasis entered into a clinical trial involving Vigil in combination with standard of care modified FOLFOX-6 chemotherapy. The first dose of Vigil was given two weeks before the modified FOLFOX-6 regimen. Vigil treatment continued until Vigil supply was exhausted. Both patients exhibited remarkable response to combination therapy, demonstrating no evidence of disease recurrence for over eight years. Additionally, both patients demonstrated systemic immune response to Vigil therapy as tested by ELISPOT.

## 1. Introduction

Approximately 25–30% of patients with colorectal cancer will develop liver metastases [1]. Overall, the five-year survival rate of metastatic colon cancer is less than 10% [2]. The five-year survival rate of patients amenable to complete resection of liver metastases ranges anywhere from 28 to 38% [3], however, only 25% of patients with liver metastases are candidates for complete resection and 60–80% will recur within two years following surgery [1].

Vigil is a triple-function immunotherapy composed of autologous tumor cells containing personalized cancer neoantigens, electroporated ex-vivo with the Vigil plasmid, which expresses the rhGMCSF (granulocyte-macrophage colony-stimulating factor) protein and a bifunctional shRNA targeting the pro-protein convertase furin which suppresses transforming growth factor beta immunosuppressive proteins, TGFβ1 and TGFβ2 [4]. Data published provide evidence of clinical response to Vigil for patients diagnosed with various types of cancer [4,5,6,7,8]. In Phase I and 2 trials of Vigil in patients with advanced cancer, mean effective target knockdown rates of the endogenous immunosuppressive transforming growth factors TGFβ1 and TGFβ2 was over 90% and the mean product expression of GMCSF had increased from below the threshold of detection of 7.8 pg/10^6^ nucleated cells to over 1000 pg/10^6^ nucleated cells from dissociated tumor tissue. Circulating mononuclear cells of patients treated with Vigil also demonstrated immune response specifically against their autologous tumor via ELISPOT assay [5,9]. Positive ELISPOT defined as ≥10 interferon response spots/10^6^ was seen in the majority of patients. Results of systemic immune response were further supported via assessment of Vigil induced circulation of CD8+ T cells and immune response profile of NanoString®. Patients demonstrating NanoString® expressive immune response related to MHC II expression and high TIS score correlated with clinical benefit (PFS, RFS, and OS) [10,11]. Clinical results in correlation with immune response also support early evidence of clinical benefit with respect to PFS, RFS, and OS, particularly involving ovarian cancer patients [5,9,10,12,13,14,15].

The following report presents two unique cases of clinical response, including potential evidence of sustained immunological memory, to Vigil, concurrent with intensive chemotherapy in high-risk patients with metastatic colorectal cancer with liver metastases that have undergone surgical resection.

## 2. Materials and Methods

### 2.1. Study Design

Two colon cancer patients (CLM-1001 and CLM-1002) with liver limited metastasis were entered into the study (CL-PTL-107 NCT01505166), after signing IRB approved consent, following complete surgical resection of liver metastasis, and treated with a modified FOLFOX-6 chemotherapy regimen started one week after 2 doses of Vigil (formerly known as FANG) consisting of 1.0 × 10^7^ cells/intradermal injection (maximum 12 injections). FOLFOX-6 consisted of oxaliplatin (85 mg/m^2^/2 h), l-leucovorin (200 mg/m^2^/2 h and fluorouracil (400 mg/m^2^ IV bolus [short-infusion] or 2400 mg/m^2^ 46 h continuous infusion) administered every 14 days (twice per cycle) for a maximum of 6 cycles. Hematologic activity, liver enzymes, and renal functions were monitored weekly as well as physical characteristics such as body weight and vital signs. CEA concentration levels in serum were also analyzed during the course of treatment. Vigil was administered from 4–8 weeks after tumor procurement via an intradermal injection every two weeks for the first two doses followed by every 28 days for subsequent doses. Per protocol, patients were allowed to receive up to 12 vaccines of Vigil, but the quantity of vaccines created was limited by the tumor tissue available for procurement. Per study protocol, treatment with Vigil was continued until progressive disease was noted using immune-related response criteria (irRC) [16] and RECIST v1.1 criteria or until all manufacturing Vigil doses were exhausted, whichever occurred first.

### 2.2. Tumor Procurement/Vigil Manufacturing

Gradalis Inc. (Carrollton, TX) manufactured the vaccine from tumors procured via surgical resection. The equivalent of a “golf-ball sized mass”, approximately 30 g in mass, was required for successful vaccine manufacture. This process has been previously described [7]. Briefly, the excised tumor collected in the surgical field was suspended in a sterile saline solution and transported to the manufacturing facility. A cell suspension of the tumor was created and transfected with the Vigil plasmid. Cells were subsequently harvested, washed, and irradiated at 10,000 cGy. These cells were aliquoted and frozen at a concentration of 1 × 10^7^ cells/mL. The media used to freeze and aliquot the cells consisted of a 10% DMSO (Bionichepharma US), 1% Human Serum Albumin (Buminate; Baxter) in a Plasma-Lyte A buffer set at a pH of 7.4 (Baxter).

## 3. Results

### 3.1. Patient CLM-1001 

Patient CLM-1001, a 50-year-old male presented with a one-month history of abdominal discomfort and significant changes in bowel movements but had not noted any abnormal bleeding and was otherwise asymptomatic. Prior to surgery, a computerized tomography (CT) in July 2012 identified a hyper-attenuating lesion within the right hepatic lobe, measuring 1.6 cm, suggestive for metastatic disease, and a circumferential wall thickening of a short segment of the sigmoid colon, measuring approximately 5.0 cm in length, which is consistent with neoplasm. No other suspicious mass lesions, fluid collections nor distention of the urinary bladder or lymphadenopathy was noted. The patient was subsequently diagnosed with Stage IVA colorectal cancer with metastatic lesions to the liver in August 2012. A subsequent colonoscopy identified a colonic lesion within the sigmoid colon and was referred for a sigmoidectomy. Pathological analysis of tissue collected during the sigmoidectomy and resection of the hepatic lesion, in September 2012, confirmed a diagnosis of moderately differentiated stage IV adenocarcinoma and operative reports dictated that 1 out of 24 pericolic lymph nodes were involved and vascular invasion was noted. Molecular testing did not detect KRAS mutation. Post-operative positron emission tomography (PET) scan did not demonstrate any changes suggestive of metastatic progression. Tumor tissue from the resected liver metastases was used to create 12 vaccines of Vigil at a concentration of 1 × 10^7^ cells/mL. Post-surgical resection, patient CLM-1001 achieved no evidential disease (NED) per Response Evaluation Criteria In Solid Tumors (RECIST) v1.1 criteria at baseline of treatment and underwent adjuvant treatment with Vigil, 8 weeks after tissue procurement as part of study CL-PTL-107, starting November 2012. In December 2012, the patient began concurrent Vigil with FOLFOX-6 therapy. The last date of treatment with FOLFOX-6 was May 2013 and the last date of Vigil administration was September 2013.

Baseline ELISPOT in November 2012, prior to treatment initiation, was measured at 0 spots/10^6^ cells (negative result). ELISPOT assays conducted during treatment Cycles 2 and 3 demonstrated ELISPOT positive results of 32 spots/10^6^ cells, and 353 spots/10^6^ cells, respectively. CEA levels remained low (<0.8 mg/dL) for over 8 years after treatment with Vigil. Follow-up visits after treatment demonstrated that patient CLM-1001 sustained an NED status per RECIST v 1.1 guidelines and the most recent follow-up visit occurred in July 2021.

During the course of treatment, Patient CLM-1001 experienced Grade 1–3 adverse events. Two adverse events of Grade 3 neutropenia and leukopenia were experienced related to FOLFOX-6. One dose modification was noted for this patient. The only toxicity developed likely in response to Vigil were two events of Grade 1 induration at the injection site. Other toxicities experienced were either unrelated to the combination regimen or were due to the FOLFOX-6 chemotherapy.

### 3.2. Patient CLM-1002 

Patient CLM-1002, a 55-year-old female presented with blood in her stools in March of 2012. Subsequent colonoscopy, completed in July 2012, identified an ulcerated circumferential 4-cm mass in the proximal rectum and distal sigmoid colon. The lesion was pathologically confirmed to be moderately differentiated adenocarcinoma with a high grade of dysplasia. A CT scan of the abdomen and pelvis in July 2012 confirmed a distal sigmoidal lesion along with adjacent lymphadenopathy in the mesentery with calcifications and a 6.4-cm lesion in the right lobe of the liver. Subsequently, the patient was diagnosed with Stage IVB Colorectal Cancer with metastatic disease to the liver in July 2012. The patient received systemic chemotherapy with FOLFOX-6 for 2 cycles (4 treatments) from July 2012 to September 2012. Following FOLFOX-6 treatment, patient CLM-1002 underwent a sigmoidectomy in October 2012 and resection of the liver metastases, which were used to prepare the autologous Vigil vaccine. From the resected liver lesions, 9 vials of Vigil at a dosage of 1 × 10^7^ cells/mL were created. Patient CLM-1002 achieved NED status per RECIST v 1.1 criteria at baseline and started treatment with Vigil 5.9 weeks after tissue procurement as part of study CL-PTL-107 in November 2012 and resumed FOLFOX-6 in December 2012. The last treatment with FOLFOX-6 occurred in March 2013, and the last treatment with Vigil occurred in June 2013.

Baseline ELISPOT prior to treatment start in November 2012 was measured at 1 spot/10^6^ cells (negative result). Repeated ELISPOT assays during cycles 1, 2, and 3 yielded results of 243 spots/10^6^ cells, 336 spots/10^6^ cells, and 150 spots/10^6^ cells (positive results), respectively. Follow-up ELISPOT assays completed 11 and 25 months after treatment initiation (4 and 7 months after treatment completion), measured 173 spots/10^6^ cells and 174 spots/10^6^ cells (positive results), respectively, with continued NED status. CEA levels up to 11.8 mg/dL were also reduced and maintained below 5.0 mg/dL (2.2–4.9 mg/dL) for over 8 years after treatment with Vigil. No recurrence has been noted in Patient CLM-1002 since treatment, with the most recent follow-up visit occurring on 17 May 2021.

Patient CLM-1002 experienced Grade 1–3 adverse events throughout the course of treatment with Vigil and FOLFOX-6. Only 1 FOLFOX-6 treatment-related Grade 3 adverse event of neutropenia was experienced. Six dose reductions of FOLFOX-6 were noted. The only adverse events likely related to Vigil were 6 Grade 1 injection site reactions (induration and erythema). The other adverse events were likely due to FOLFOX-6.

## 4. Discussion

In summary, we present two patients that had advanced colorectal cancer with completely resected liver metastases who sustained remarkable NED status for over eight years (CLM-1001 over eight years six months and CLM-1002 over eight years nine months) after treatment with the Vigil vaccine and concurrent chemotherapy with FOLFOX-6. Moreover, both demonstrated evidence of active immune response stimulation against their own colon cancer cells as tested via ELISPOT reactivity.

The maintained NED statuses seen in both patients after Vigil treatment as well as correlative ELISPOT results support durable immune-related benefit greater than expected with the standard of care. Additionally, these results were achieved without significant toxicities to a regimen of Vigil in conjunction with chemotherapy, which suggests that this course of treatment could prove to be beneficial for other colorectal cancer patients with surgically resectable liver limited metastasis.

The increase in ELISPOT assay results of both patients during the treatment period also is suggestive of a strengthened acquisition and retention of CD8+ and memory T-cells. Induction of CD8+ T cell gamma interferon with exposure to relevant clonal tumor neoantigens has been defined as a significant mechanism related to mononuclear cell induced antitumor immune response [17]. Such a mechanism is postulated to be a critical component of the immune response associated with RFS and OS benefit involving several studies with Vigil [5,7,8,9,12,13,14]. Molecular signal assessment may facilitate identification of resistance and/or sensitivity to Vigil therapy. Bone marrow derived T cell progenitor maturation involves thymus modulation and interaction of the T cell receptor with major histocompatibility complex expressed on thymic stromal cells [18]. Through this interaction, necessary CD8+, CD4+, and dual CD8+/CD4+ T cells differentiate and potentially interact with Vigil stimulation. Limits in T cell progenitor development may reduce Vigil responsiveness. Further assessment of molecular profile in patients undergoing Vigil therapy may identify optimal responsive subgroups of patients. Potential targets may include Runx3, Twist 2, Nur77, and NorI [19,20]. Ongoing work is underway. ELISPOT results have been exhaustively documented to be an indicator of systemic immune response and have support in the correlation of clinical benefit with other solid tumors [4,5,6,7,8]. These results support further investigation of the combination Vigil + FOLFOX-6 regimen to determine if the combination is effective in preventing recurrence of colorectal cancer. 

In conclusion, we presented two colorectal patients with resectable liver metastasis who had durable response (over eight years) to Vigil. Both patients exhibited systemic immune activation, which may correlate with CD8+ and memory T cell retention and generation.

## Data Availability

Data is available upon written approved request for a specific research question. Proposals and requests that pose a competitive risk may be declined.
